# Forecasting Seasonal *Vibrio parahaemolyticus* Concentrations in New England Shellfish

**DOI:** 10.3390/ijerph16224341

**Published:** 2019-11-07

**Authors:** Meghan A. Hartwick, Erin A. Urquhart, Cheryl A. Whistler, Vaughn S. Cooper, Elena N. Naumova, Stephen H. Jones

**Affiliations:** 1 Northeast Center for Vibrio Disease and Ecology, University of New Hampshire, Durham, NH 03824, USA; mah2002@wildcats.unh.edu (M.A.H.); Urquhart.erin@epa.gov (E.A.U.); Cheryl.Whistler@unh.edu (C.A.W.); 2 Department of Molecular, Cellular, and Biomedical Sciences, University of New Hampshire, Durham, NH 03824, USA; 3 Department of Natural Resources and the Environment, University of New Hampshire, Durham, NH 03824, USA; 4 Department of Microbiology and Molecular Genetics, University of Pittsburgh School of Medicine, Pittsburgh, PA 15261, USA; vaughn.cooper@pitt.edu; 5 Division of Nutrition Data Sciences, Friedman School of Nutrition Science and Policy, Tufts University, Boston, MA 02111, USA; elena.naumova@tufts.edu

**Keywords:** *Vibrio parahaemolyticus*, seasonality, seafood illness, forecasting, climate change

## Abstract

Seafood-borne *Vibrio parahaemolyticus* illness is a global public health issue facing resource managers and the seafood industry. The recent increase in shellfish-borne illnesses in the Northeast United States has resulted in the application of intensive management practices based on a limited understanding of when and where risks are present. We aim to determine the contribution of factors that affect *V. parahaemolyticus* concentrations in oysters (*Crassostrea virginica*) using ten years of surveillance data for environmental and climate conditions in the Great Bay Estuary of New Hampshire from 2007 to 2016. A time series analysis was applied to analyze *V. parahaemolyticus* concentrations and local environmental predictors and develop predictive models. Whereas many environmental variables correlated with *V. parahaemolyticus* concentrations, only a few retained significance in capturing trends, seasonality and data variability. The optimal predictive model contained water temperature and pH, photoperiod, and the calendar day of study. The model enabled relatively accurate seasonality-based prediction of *V. parahaemolyticus* concentrations for 2014–2016 based on the 2007–2013 dataset and captured the increasing trend in extreme values of *V. parahaemolyticus* concentrations. The developed method enables the informative tracking of *V. parahaemolyticus* concentrations in coastal ecosystems and presents a useful platform for developing area-specific risk forecasting models.

## 1. Introduction

*Vibrio parahaemolyticus* is the leading cause of seafood-borne gastroenteritis in the US and worldwide [1,2,3]. Most strains are believed to be non-pathogenic and the strains that do cause gastroenteritis and septicemia in humans have been historically associated with warm water environments [4,5,6]. Over the past decade, however, illnesses caused by *V. parahaemolyticus* have become more frequent in some cold and temperate water environments where illnesses were previously rare [7,8,9,10,11,12,13,14]. This new pattern of *V. parahaemolyticus* disease likely stems from a combination of observed trends, such as introduced and ecosystem establishment of pathogenic strains, increased summertime production and consumption of raw shellfish, and climate related changes causing warmer sea surface temperatures and more variable salinities [7,8,13,14,15,16,17,18,19]. In the Northeast United States (US) where pathogenic *V. parahaemolyticus* is now established, foodborne illness is most frequently acquired from the consumption of raw or undercooked shellfish [3]. Post-harvest management has effectively reduced the incidence of *V. parahaemolyticus* disease outbreaks in this region. However, illness still occurs and achieving effective post-harvest control is both resource and time intensive. Effective pre-harvest *V. parahaemolyticus* forecasting tools would be valuable to shellfish growers and managers alike to make informed decisions about the *V. parahaemolyticus* risk conditions at the time of harvest and potentially reduce the risk and cost of *V. parahaemolyticus* management. 

*V. parahaemolyticus* is a naturally occurring bacterial species that persists in a wide range of conditions in most marine and estuarine environments [5,20,21,22,23,24,25,26,27,28,29,30]. In multiple studies, temperature and salinity correlate most strongly with *V. parahaemolyticus*, but the strength of this relationship varies by region and season [31]. Similarly, nutrients, chlorophyll *a*, pH and turbidity were inconsistent and depended on the region and the variability of these factors. Therefore, region and even harvest area-specific studies are necessary to provide an accurate description of the influence of environmental conditions on *V. parahaemolyticus* concentration [32]. 

**Figure 1:**
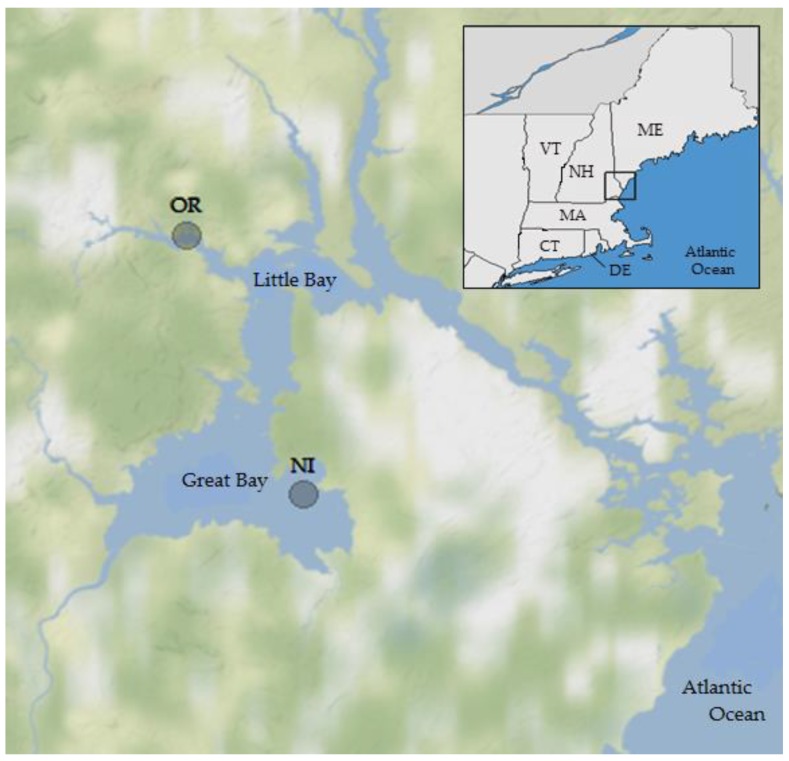
Study area and sites for oyster and water sampling in the Great Bay Estuary, New Hampshire, USA. OR = Oyster River; NI = Nannie Island.

Long-term monitoring has been established in the Great Bay Estuary (GBE) by the Northeast Center for *Vibrio* Disease and Ecology at the University of New Hampshire (UNH) since 2007 [33,34,35,36]. The GBE is located on the border of New Hampshire and Maine (Figure 1) and has a long history of studies on pathogenic *Vibrio spp*. [37,38,39]. It is a regionally significant estuary that experiences wide-ranging environmental, climatic, and biological conditions [10], and thus serves as a useful model representative of regional estuaries. It is unique in that *V. parahaemolyticus* illnesses are still rare [40], although the *V. parahaemolyticus* population in the Northeast is evolving [13,14] and commercial shellfish harvests are rapidly increasing. The ongoing surveillance enables the development of pre-harvest risk-forecasting models. 

The goal of this study was to develop an integrated modeling approach to predict *V. parahaemolyticus* concentrations in shellfish at a pre-harvesting stage as a tool for managing this significant public health issue. We used data from 2007 to 2016 to capture long-term trends, seasonal fluctuations in a broad range of environmental and climatic predictors of *V. parahaemolyticus* dynamics, aiming to create a model development approach that could be transferable to other estuaries.

## 2. Materials and Methods

### 2.1. Study Sites, Environmental Sampling and Bacterial Analysis

The study area was the Great Bay estuary in New Hampshire. The two sampling locations (Figure 1) were near Nannie Island (NI) in Great Bay, where shellfish harvest classification is approved, and a site in the tidal portion of the Oyster River (OR), where harvesting is prohibited because of proximity to the Durham NH wastewater treatment facility. Both are locations of significant oyster (*Crassostrea virginica*) beds and long-term monitoring locations [36,38] and have different ecosystem and environmental conditions. The estuary has been monitored for over 30 consecutive years (March–December) through efforts by multiple agencies, including the Great Bay National Estuarine Research Reserve (GBNERR) and their System Wide Monitoring Program [41]. The average temperature, salinity, dissolved oxygen (DO), pH, and turbidity data were calculated from continuous (Q15) measurements obtained from the SWMP database for 2007–2016 for times simultaneous with and preceding oyster sampling in 12 h periods to account for ecological lag times and capture a more complete assessment of the potential environmental conditions that may have contributed *V. parahaemolyticus* concentrations observed at the time of collection. Monthly SWMP samples provided nutrient (total dissolved nitrogen (TDN)) and chlorophyll *a* (concentration by fluorescence; CHL)) data for monitoring sites in close proximity to the NI and OR sampling locations. Meteorological data were acquired from several weather stations (SWMP; UNH) in the Great Bay region. Water quality parameters were measured in situ at the time of sampling using calibrated YSI 6600 and EXO multiprobe datasondes (Yellow Springs Instruments, Yellow Springs, OH, USA). 

### 2.2. Oyster Sample Collection and Processing

Oyster samples were collected from the two oyster beds at NI and OR except during the period January–March from June 2007 through December 2016. For each sampling date, 10–12 oysters were cleaned and aseptically shucked into a sterile beaker (liquor and meat), weighed and diluted 1:1 with alkaline peptone water (APW (pH 8.6, 1% NaCl), and homogenized. A volume of 20 mL homogenate was further diluted in 80 mL APW for a starting dilution of 1:10. A volume of 1 mL of 1:10 solution was added to three tubes and then serially diluted with 1 mL aliquots into three serial dilutions containing 9 mL APW (pH 8.6, 1% NaCl). Each tube was incubated at 37 °C overnight (18–20 h) following the U.S. Food and Drug Administration Bacteriological Analytical Manual (BAM) [42]. 

Following incubation, turbid APW tubes were scored positive for growth. From 2007 to 2010, turbid tubes were streaked to Thioglycollate-Citrate-Bile-Salt (TCBS) agar (Beckton Dickson (BD), Franklin Lakes, NJ, USA) and incubated at 37 °C for 18–20 h. From 2011 to 2016, turbid tubes were streaked onto Vibrio CHROMAgar (CHROMagar, Paris, France) and incubated at 37 °C for 18–20 h. Sucrose negative (green) colonies from TCBS or purple colonies from CHROMagar were streaked onto tryptic soy agar (TSA; BD) and incubated at room temperature for 18–20 h. TSA isolates were inoculated in Heart Infusion (HI) broth for 18–20 h. Then, 1 mL HI aliquots were pelleted for 5 min at 8000 rpm, re-suspended in 1 mL molecular biology grade water (Phenix Research Products, Candler, NC, USA), boiled at 100 °C for 10 min and debris removed by centrifugation. Species identity of isolates was determined by polymerase chain reaction (PCR) performed using 2 μL cleared supernatant in 13 μL Mastermix, iQSupermix (BioRad, Hercules, CA, USA) using a BIO RAD T100 thermocycler and published primers and conditions [43] for 2007–2014, with slight modifications for 2015–2016 [19]. The PCR amplicons were visualized on 1.2% agarose gel with addition of Gel Red (Phenix Research Products, Candler, NC, USA) under UV light. The concentration (Most Probable Number) was calculated from *V. parahaemolyticus* species-specific gene (tlh)-confirmed isolates from enrichment tubes and the BAM Most Probable Number (MPN) tables.

### 2.3. Statistical Analysis

All statistical computations were performed in the R Statistical Program and Environment, version 3.5.1 [44] with add-on packages MGCV [45]. Graphics were produced with ggplot2 [46]. Multiple steps of data analysis were performed to evaluate the relationship between environmental determinants, seasonality and *V. parahaemolyticus* concentrations in the GBE. MPN values for *V. parahaemolyticus* concentrations were log-transformed for analysis and model development to approximate normality and reduce skewness. Sampling events with missing environmental measurements (*n* = 29) when *V. parahaemolyticus* was not detected (*n* = 71) and one sample that exceeded >2 standard deviations were excluded from concentration model development. Statistical significance for all analysis in this study was determined using an alpha level of *p* < 0.05.

#### 2.3.1. Model Development Strategy

All measurements were arranged in chronological order based on the date of measurement and multiple time series were compiled for the entire study period. The relationships between the time series for water quality variables, including water temperature, salinity, pH, DO, turbidity, CHL, TDN, rainfall and *V. parahaemolyticus* concentrations in oysters were evaluated using correlation and regression analysis. We used log-transformed values of *V. parahaemolyticus* concentrations (*Y_t_*) and applied a Gaussian family distribution with an identity link function relating the expected value of response variable *Y_t_* to selected predictors [47,48]. The transformation of water temperature, salinity, pH, DO, turbidity, CHL, TDN and rainfall was also explored as response variables in seasonality analysis and as predictor variables for *V. parahaemolyticus* in regression analysis with log or log + 1. We assessed the shape of relationships (linear and non-linear) between *V. parahaemolyticus* concentrations in oysters and environmental predictors. Variables that were significant in univariate regression were used to develop multiple regression models. We also assessed seasonality and trends over time and explored alternative variables representing seasonality with respect to their ability to improve the stability of forecasting. Assumptions of inter-correlation among predictors were evaluated using Spearman correlation analysis. Below we provide the detailed description of model building.

#### 2.3.2. Seasonality and Trend Analysis

To explore the seasonality and the general trend throughout the whole study period (2007–2016) in all variables—*V. parahaemolyticus* concentrations, temperature, DO, salinity, pH, turbidity, CHL, TDN and rainfall—we developed two models with different ways of presenting the periodicity of seasonal oscillations. Model 1 contains variables for a linear trend and photoperiod. Model 2 uses terms for a linear trend and harmonic regression terms for the calendar day in the study as follows:

(1) $$\mathrm{Model}1:E\left(Y_{t}\right)=\beta_{0}+\beta_{1}t+\beta_{p}Photoperiod,$$

(2) $$\mathrm{Model}2:E\left(Y_{t}\right)=\beta_{0}+\beta_{1}t+\beta_{s}\sin\left(2\pi \omega t\right)+\beta_{c}\cos\left(2\pi \omega t\right).$$

In both models, $Y_{t}$ is the daily time series for the outcome of interest, *β*_0_ is the intercept, *t* is the daily time series, $\beta_{1}$ indicated a general trend in the outcome of interest, *β_s_* and *β_c_* are the coefficients of the harmonic terms and ω is the term representing the annual cycle (365.25 days, ω = 1 / 365.25). The harmonic terms in Model 2 are expected to depict the periodic oscillation that can also be captured by the *β*_p_ in Model 1. The phase shift of periodic oscillations identified by Model 2 was determined as follows:

(3) $$\psi =\arctan\left(\frac{\hat{\beta }s}{\hat{\beta }c}\right)+k.$$

When estimates of ${\hat{\beta }}_{S}$ and ${\hat{\beta }}_{c}$ were positive, k = 0. If ${\hat{\beta }}_{S}<0$ and ${\hat{\beta }}_{c}>0,$ then k=2π. If ${\hat{\beta }}_{S}$ and ${\hat{\beta }}_{c}$ were negative, or if ${\hat{\beta }}_{S}>0$ and ${\hat{\beta }}_{c}<0,$ then k=π. The phase shift (ψ) was multiplied by 365.25 days in order to calculate peak timing. Covariance of $\beta_{s}$ and $\beta_{c}$ ($\sigma_{\beta_{c}\beta_{s}})$ and variance of $\beta_{s}$ and $\beta_{c}$ (${\hat{\beta }}_{s}^{2}$ and ${\hat{\beta }}_{c}^{2})$ estimated the variance of the phase shift (ψ) as:

(4) $$var\left(\psi \right)=\frac{{\left(\sigma_{\beta s}\beta c\right)}^{2}+{\left(\sigma_{\beta c}\beta s\right)}^{2}-\left(2\sigma_{\beta_{c}\beta_{s}}\beta_{s}\beta_{c}\right)}{\left({\hat{\beta }}_{s}^{2}+{\hat{\beta }}_{c}^{2}\right){\left({\hat{\beta }}_{s}^{2}+{\hat{\beta }}_{c}^{2}\right)}^{2}},$$

and confidence intervals of the peak timing were determined as: $1.96\times \sqrt{Var\left(\psi \right)\times 365.25/2\pi }.$ Secular trends were assessed using nine default thin-plate splines (f) from the MGCV package in R [46] in Model 3 and Model 4 as shown

(5) $$\mathrm{Model}3:E\left(Y_{t}\right)=\beta_{0}+\beta_{1}f\left(t\right)+\beta_{p}Photoperiod,$$

(6) $$\mathrm{Model}4:E\left(Y_{t}\right)=\beta_{0}+\beta_{1}f\left(t\right)+\beta_{s}sin\left(2\pi \omega t\right)+\beta_{c}cos\left(2\pi \omega t\right).$$

The models’ performance was determined by the deviance explained, residual variation, Akaike’s Information Criterion (AIC), and coefficient of determination (*r*^2^) value. The trend term was determined to be non-linear based on visual assessment, positive ∆AIC and positive ∆*r*^2^ and ∆Deviance > 0.1.

#### 2.3.3. Extreme Value Trend Analysis

In addition to a general trend and Mann–Kendall trend analysis, we explored potential trends in high values of *V. parahaemolyticus* concentration as well as TDN, pH and salinity based on their importance in multiple regression models to estimate *V. parahaemolyticus* concentration by determining the number of events when the observations were above its 75th percentile. For other variables, trends were evaluated using the number of observations within the 25th and 75th percentile.

#### 2.3.4. Variable Selection and Non-Linearity Assessment

To explore the relationship between the response variable, *V. parahaemolyticus* concentrations, and predictor variables, we incorporated each environmental parameter individually into linear (Model 5) and non-linear (Model 6) regression models. These two models were applied to the log-transformed values of *V. parahaemolyticus* concentrations (*Y_t_*):

(7) $$\mathrm{Model}5:E\left(Y_{t}\right)=\beta_{0}+\beta_{1}X_{t},$$

where $Y_{t}$ is the daily time series for the log-transformed *V. parahaemolyticus* concentrations in oyster, $X_{t}$ is the daily time series for an environmental predictor and $\beta_{1}$ reflects the degree of captured linear relation in the daily time series of response and predictor variables. 

Non-linear relationships were initially assessed using nine default thin-plate splines (f) from the MGCV package in R [46] as shown in

(8) $$\mathrm{Model}6:E\left(Y_{t}\right)=\beta_{0}+\beta_{1}f(X_{t}).$$

The relationships between the environmental conditions and *V. parahaemolyticus* concentrations were overlaid with loess curves to visualize the relationship. Non-linear relationships were evaluated by the differences between the significance of the coefficient, residual variation, AIC, and coefficient of determination (*r*^2^) value. Positive values indicate that the measure improved in Model 6 compared to Model 5 and negative values indicate a decrease in the model evaluation measurement. Variables were determined to be non-linear based on visual assessment, positive ΔAIC and positive $\Delta r^{2}$ and ΔDeviance > 0.1. When strong non-linear non-monotonic relationships were detected, we re-parametrized the predictor by centering the variable around its *V. parahaemolyticus* concentration maximum and created a new variable to provide biological interpretability to the model [49]. For example, a new variable for pH was created by squaring the difference between the observed pH values and the value of 7.8 selected for the centering. Re-parametrized variables are indicated as C-variable name (e.g., C-pH). 

#### 2.3.5. Model Building

The environmental parameters determined to be significant in univariate models (Models 5 and 6) were incorporated into a multivariate general linear regression model using Gaussian (GLM-G) and negative binomial (GLM-NB) distributional assumptions. For GLM-NB, the dispersion was determined by the index of dispersion: ∅ = *variance*/*mean* = 1, where ∅ < 1 refers to under-dispersion and ∅>1 refers to over-dispersion. We started with the sequential model building (Model 7):

(9) $$\mathrm{Model}7:E\left(Y_{t}\right)=\beta_{0}+\beta_{1}X_{1,t}+\beta_{k}X_{k,t},$$

where $Y_{t}$ is the daily time series for the outcome of interest, *β*_0_ is the intercept and *t* is the daily time series; $X_{1,t}\ldots X_{k,t}$ are the daily time series for environmental predictors, including the reparametrized centered variables and interaction terms; $\beta_{1}\ldots \beta_{k}$ are the corresponding coefficients. 

We then added variables to reflect the trend and seasonal oscillations and fine-tuned the model by using the photoperiod variable (Model 8), or harmonic terms (Model 9). In both models:

(10) $$\mathrm{Model}8:E\left(Y_{t}\right)=\beta_{0}+\beta_{1}X_{1,t}+\beta_{2}X_{2,t}+\ldots \beta_{l}t+\beta_{p}Photoperiod,$$

(11) $$\mathrm{Model}9:E\left(Y_{t}\right)=\beta_{0}+\beta_{1}X_{1,t}+\beta_{2}X_{2,t}+\ldots \beta_{l}t+\beta_{s}\sin\left(2\pi \omega t\right)+\beta_{c}\cos\left(2\pi \omega t\right),$$

where $Y_{t}$ is the daily time series for the outcome of interest, *β*_0_ is the intercept and *t* is the daily time series; $X_{1,t}\ldots X_{k,t}$ are the daily time series for the selected environmental predictors, including the reparametrized centered variables and interaction terms; $\beta_{1}\ldots \beta_{l}$ are the corresponding coefficients. In Model 8 and 9, $\beta_{p}$ is the coefficient of the photoperiod variable. In Model 10, *β_s_* and *β_c_* are the coefficients of the harmonic terms and ω is the term representing the annual cycle (365.25 days), as in Model 2. 

For these hybrid models, we employed sequential model building using both Gaussian and negative binomial distributional assumptions in parallel and explored the contribution of interaction terms to the model’s fit. Overall performance of GLMs was evaluated by evaluation of Akaike’s Information Criterion (AIC) [50], residual variation, and deviance explained to determine the number and combination of variables that provided the strongest fit for the full time period of 2007–2016. Model fit was evaluated by the differences between the significance of the coefficient, residual variation, AIC, and coefficient of determination (*r*^2^) value. Model selection was based on AIC value and improvement of $r^{2}$ and deviance explained >0.1.

Using the parameters of the harmonic terms, e.g., the estimates of $\beta_{s}$ and $\beta_{c}$ regression coefficients and their error from Model 9, we applied the δ-method [51,52] to estimate seasonal peak timing along with its error term, expressed in days. 

### 2.4. Assessment of Model Forecasting Ability

The predictive skill or forecasting ability of the selected versions of Models 7, 8 and 9 were evaluated by splitting the whole dataset into two datasets representing two periods: a training dataset from 2007 to 2013, and a test dataset from 2014 to 2016. Correlations between environmental variables and *V. parahaemolyticus* concentrations were compared for the full, training, and testing intervals. The forecasting ability and model performance were determined by coefficient of determination (*r*^2^), and overall residual deviance. Forecasting error was evaluated by root mean square error (RMSE).

## 3. Results

### 3.1. V. parahaemolyticus Concentrations in the GBE, 2007–2016

**Figure 2:**
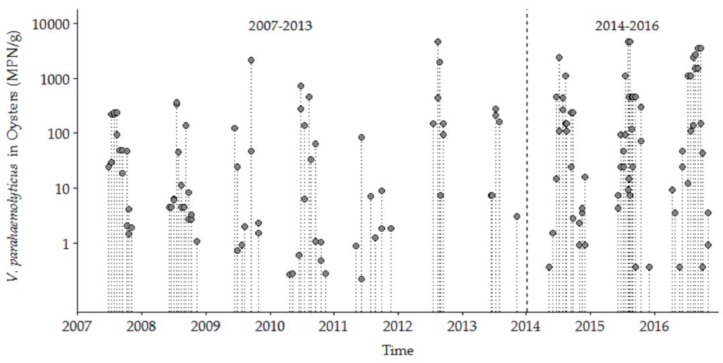
*Vibrio parahaemolyticus* concentrations in oysters from NI and OR at low tide in the Great Bay Estuary (GBE) in 2007–2016.

*V. parahaemolyticus* was detected in 144 oyster samples during May through December from 27 June 2007 to 5 December 2016 for both the NI (*n* = 77) and OR (*n* = 67) study sites that included complete sets of data for environmental variables. There were no significant between-site differences for *V. parahaemolyticus* concentrations or measured condition parameters (data not shown) at the two sites, so all the following analyses use a combined-site database. Our analysis of the samples from 2007 to 2016 included the detection of *tdh* and *trh*, the traditional indicator markers for presence of ‘pathogenic’ *V. parahaemolyticus*. However, these markers were only detected in two samples during 2009 and were not detected again until 2015. Thus, our study focused on total *V. parahaemolyticus* concentrations because it is important to understand the ecosystem dynamics of the population of this species in shellfish harvest areas [36] as a proxy for risk assessment, and, in part because not all *V. parahaemolyticus* strains in clinical cases in the Northeast US and elsewhere contain either *tdh* or *trh*. Three main aspects of the full ten-year database are the marked seasonality, upward trend in high concentration values, and the wide variability/dispersion of *V. parahaemolyticus* concentrations between years and within each year (Figure 2). The observed *V. parahaemolyticus* concentrations were highly seasonal, ranging from 0.036 MPN/g oyster tissue during cold seasonal conditions to 4600 MPN/g during warm summertime conditions. The highest annual *V. parahaemolyticus* concentrations were higher during the later years than in early years. The detailed analysis of the trends and seasonality is presented below. 

#### 3.1.1. Trends and Seasonality

*V. parahaemolyticus* concentrations in oysters and environmental variables in the GBE were formally assessed for seasonality by using a photoperiod (Model 1) and a harmonic regression model (Model 2). These models allowed us to determine whether the study variables displayed re-occurring periodicity and a linear trend using calendar day of study to assess change over time. We examined trends, peak timing and seasonal oscillations in water temperature, DO, salinity, pH, turbidity, CHL, TDN and rainfall. The patterns of data had various shapes, including an extended period of *V. parahaemolyticus* detection during fall compared to spring. The variability in *V. parahaemolyticus* concentration in oysters, water temperature, DO and salinity were highly seasonal and well detected by both photoperiod and harmonic regression models, though the harmonic regression model provided a better fit in all instances (Table 1). 

**Figure 3:**
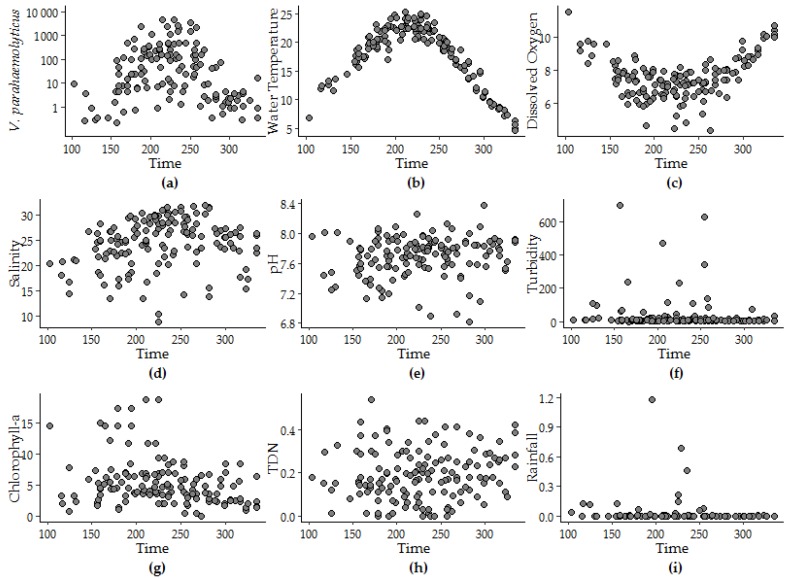
Patterns in (**a**) *V. parahaemolyticus* concentration, (**b**) water temperature, (**c**) dissolved oxygen, (**d**) salinity, (**e**) pH, (**f**) turbidity, (**g**) CHL, (**h**) TDN, and (**i**) rainfall versus the calendar day of the year superimposed from 2007 to 2016.

The peak timing of *V. parahaemolyticus* (day 222 ± 5) was determined to be approximately 10 days after the peak timing of water temperature that occurred at day 213 ± 2. The peak timing of salinity and pH were within 25 days of the peak timing of *V. parahaemolyticus*, though as the strength of the seasonality of the variable decreased the confidence interval around the corresponding the peak timing was observed to increase. Neither model offered a fit to the variability observed in other variables. For instance, less than 4% of the variability in rainfall was attributed to seasonality. Rainfall and turbidity measurements above zero were episodic and model fit did not improve above 1% variance explained with log or log + 1 transformation. Figure 3 provides an explanation for the model fit by superimposing daily values for each year and depicting seasonal patterns for each variable.

**Figure 4:**
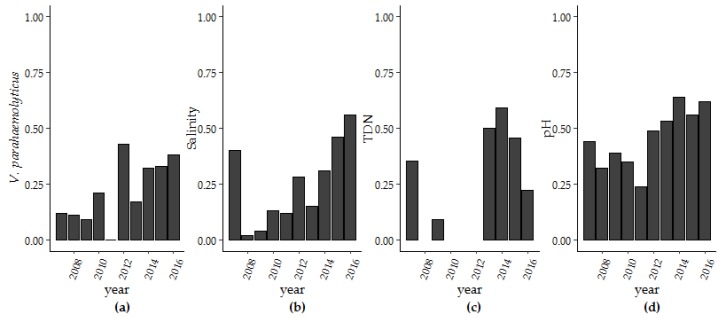
The number of observations per year above the 75th percentile for (**a**) *V. parahaemolyticus* concentrations, (**b**) salinity, (**c**) TDN and between the 25th and 75th percentile for (**d**) pH.

Over the ten-year period of surveillance there were significant increases in *V. parahaemolyticus* concentrations, salinity, pH and TDN. Only turbidity decreased during this same period (Table 1). To further explore these findings, we used thin-plate splines to assess secular trends (Table S1) and examined the trend in extreme values for salinity, pH, TDN, and *V. parahaemolyticus* concentrations that were above the 75% percentile (Table 2; Figure 4). The improvement in fit from the non-linear trend term in the photoperiod model (Model 1 and Model 3) was not seen in the harmonic regression model (Model 2 and Model 4). The change in fit can be largely attributed to interannual variation that was accounted for in the harmonic regression model and so a linear trend term was applied moving forward. For pH, the range of observed pH values decreased over time with more observations occurring within the pH range of 7.56 (25th percentile) to 7.88 (75th percentile). For salinity and pH, more than 55% and 62.1% of days observed in 2015 were above 27.0 ppt for salinity and within the 7.56–7.88 pH range, respectively. TDN above 0.27 mg/L was observed in in six of ten years of the study. In later years, at least 45.0% percent of measured TDN was above the 75% percentile. Yearly *V. parahaemolyticus* concentrations above 240 MPN/g oyster tissue increased from 11.8% of samples in 2007 to 38.1% in 2016. Kendall-Mann trend analysis identified significant upward trends in extreme values for *V. parahaemolyticus*, salinity, pH, and TDN (*p* < 0.05).

#### 3.1.2. Univariate Regression

Individual linear and non-linear regression analyses conducted between *V. parahaemolyticus* concentration in oysters and eight measured variables from 2007 to 2016 identified water temperature, salinity, DO, pH, CHL and rainfall as significant model parameters in linear or non-linear regression. Model fit improved by less than 0.1 with log or log + 1 transformation of the independent variables. Water temperature accounted for the largest degree of *V. parahaemolyticus* variation (48.1%), DO accounted for 32.1%, followed by salinity (11.0%), pH (4.8%), CHL (2.8%) and rainfall (2.3%). The significance of pH increased in non-linear versus linear regression (Table 3), and the variability explained by pH also increased from 4.8% to 13.4%. 

**Figure 5:**
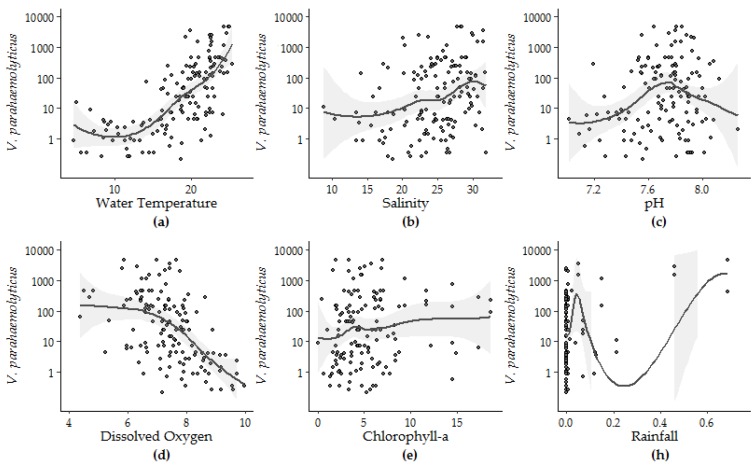
Loess smoothing applied to *V. parahaemolyticus* concentrations and (**a**) water temperature, (**b**) salinity, (**c**) pH, (**d**) DO—dissolved oxygen, (**e**) CHL—chlorophyll-*a*, and (**h**) rainfall.

The form of the relationship between the environmental conditions and *V. parahaemolyticus* concentrations was further explored using loess smoothing to determine the parameters for each variable (Figure 5). The strength and significance of the linear response (Model 5) can be observed between *V. parahaemolyticus* and temperature, salinity and DO. Likewise, the non-linear relationship between pH and *V. parahaemolyticus,* identified by Model 6, is also highlighted by the loess smoothing. Though the fit between *V. parahaemolyticus* and CHL improved in Model 4 compared to Model 3, visual inspection of this relationship shows that this improvement can be attributed to rare events in the extremes of the observations.

The nonlinear regression between pH and *V. parahaemolyticus* was first improved with the addition of thin-plate splines. Based on the application of loess smoothing, pH was then re-parametrized as the square of the difference between the observed pH and 7.8, an apparent ecological optimum relative to observed *V. parahaemolyticus* concentrations in the study area. Re-parameterization of pH improved the percent variability explained, *r*^2^ and *p* values from 4.8%, 0.04 and 0.008 (for the unmodified pH data) to 8.6%, 0.1 and 0.0003, respectively.

### 3.2. Sequential Model Building

**Figure 6:**
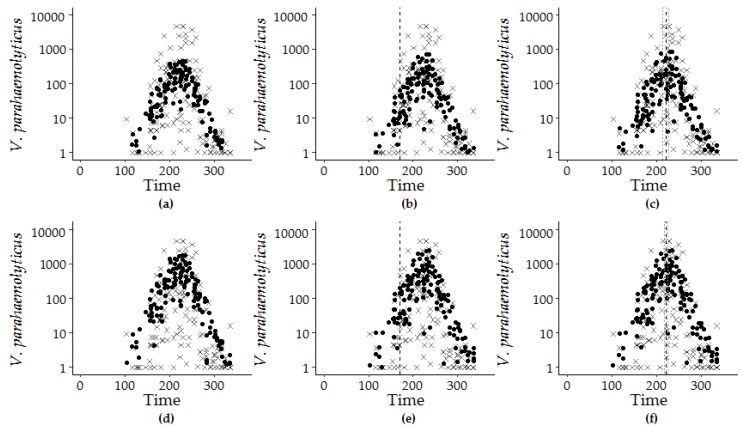
Model estimations (filled circle) and observed *V. parahaemolyticus* concentrations (x) are superimposed by the calendar day of the year from 2007 to 2016: GLM-G for (**a**) Model 7.4, (**b**) Model 8.1, and (**c**) Model 9.1 and GLM-NB for (**d**) Model 7.4, (**e**) Model 8.1, and (**f**) Model 9.1. The dashed vertical line at day 170 for the hybrid model (**b**,**e**) marks the longest day of the year, and the dashed lines at day 222 ± 5 days and at day 221 ± 7 days indicate the calculated peak timing of *V. parahaemolyticus* concentration for Model 7.1 for (**c**) GLM-G and (**f**) GLM-NB versions.

A multiple regression model was next developed to determine a set of environmental variables that predict *V. parahaemolyticus* concentrations in oysters between 2007 and 2016 (Table 4). Water temperature was a foundational model variable for multiple regression model development and thus used in all multiple variable regression models. Single and multi-parameter models excluding water temperature explained less than the 48.1% of *V. parahaemolyticus* concentration variation explained by water temperature alone (data not shown). The addition of the trend term, photoperiod and harmonic regression variables to the environmental variables in negative binomial regression optimized model estimations (Figure 6, Table S2). 

**Figure 7:**
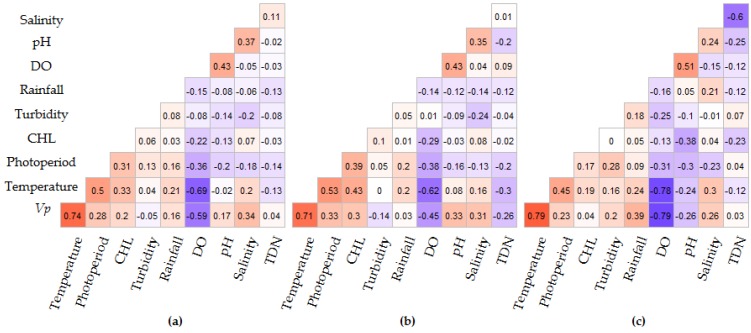
Spearman correlation analysis of *V. parahaemolyticus* concentrations and environmental variables for three intervals: (**a**) 2007–2016, (**b**) 2007–2013 and (**c**) 2014–2016. Red indicates positive and blue negative correlations and the degree of significance is highlighted by color intensity.

Spearman rank correlation analysis of the individual intervals indicates that photoperiod, water temperature, DO, pH and salinity were significantly correlated with *V. parahaemolyticus* concentrations in all time intervals, though the correlation between pH and *V. parahaemolyticus* varied between intervals (Figure 7). Inter-variable correlations were observed between water temperature and DO (R = −0.69, *p* < 0.0001), salinity (R = 0.20, *p* = 0.014), CHL (R = 0.21, *p* ≤ 0.0001) and rainfall (R = 0.21, *p* = 0.015). pH and DO (R = 0.43, *p* < 0.0001) and pH and salinity (R = 0.37, *p* < 0.0001) were also correlated. Rainfall was only significant in the test dataset, while CHL was significant in the entire and training dataset (2007–2013) intervals but not significant in the test dataset (2014–2016) interval. Significant associations were observed between photoperiod and *V. parahaemolyticus* concentrations, water temperature, DO, salinity, CHL and pH (R = 0.28, *p* ≤ 0.001; R = 0.5, *p* ≤ 0.001; R = −0.36, *p* ≤ 0.001, R = 0.18, *p* = 0.04; R = 0.33, *p* = 0.001; R = −0.20, *p* = 0.02 respectively) (Figure 7). Of the nine variables considered, only turbidity, TDN and rainfall were not correlated to *V. parahaemolyticus* concentrations or photoperiod. 

### 3.3. Model Performance Prediction

The hybrid model (Model 9.1) provided the best overall fit for each dataset time interval, with consistently lower RMSE and higher *r*^2^ values compared to the harmonic regression (Model 10.1) and environmental model (Model 7.4) (Table 5). The fits for all three models were relatively consistent even though the significance of some variables changed between time intervals. Although the estimations of precision for the harmonic regression model across training/test datasets were slightly lower than for other models, it is advantageous because important attributes of the data can be identified. For example, the *V. parahaemolyticus* concentrations peaked on 222 ± 5 day of the 365.25-day period for all three intervals. Similarly, the peak timing of water temperature and salinity were stable between the overall, training and test datasets (212 ± 2 day and 251 ± 18 day, respectively).

**Figure 8:**
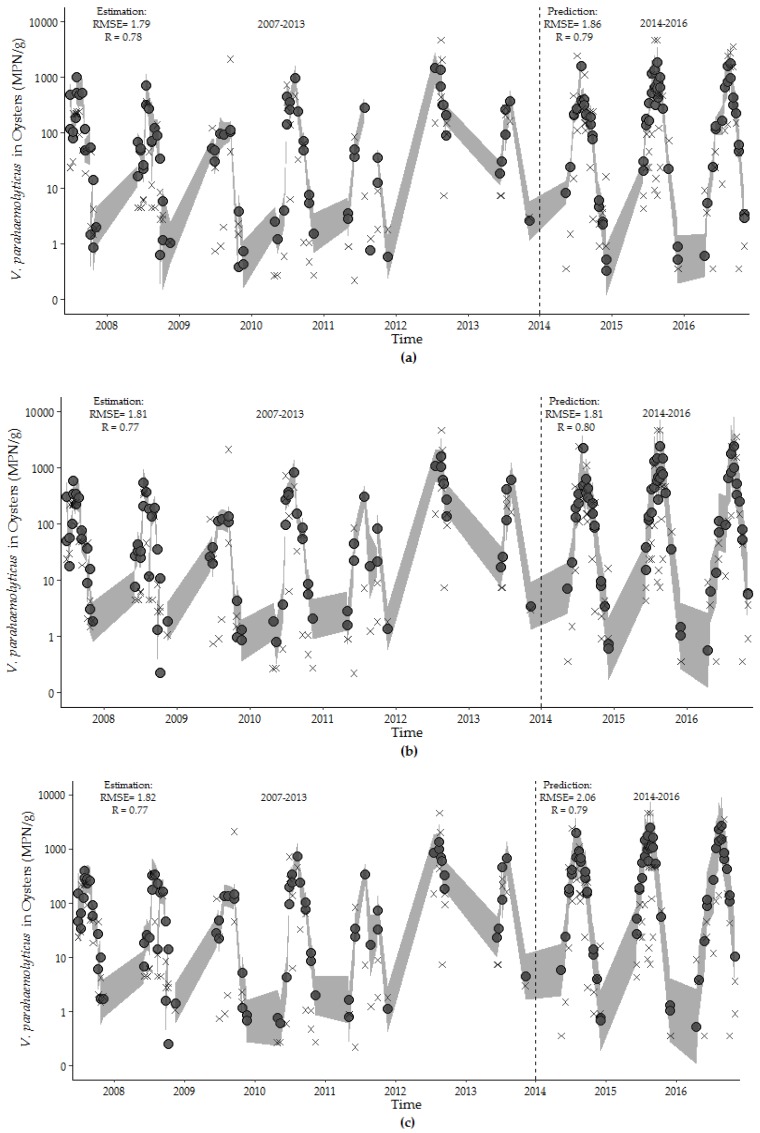
Estimates of *V. parahaemolyticus* concentrations (closed circle) with observed *V. parahaemolyticus* concentrations for: (**a**) environmental model, (**b**) hybrid model, and (**c**) harmonic regression model for the training (2007–2013) and test (2014–2016) periods. The 95th percentile prediction interval is represented by the gray shading. Model fit values are shown in the upper left corner of each figure.

The environmental (Model 7.4), hybrid (Model 8.1), and harmonic regression (Model 9.1) models developed with the observations from the training dataset accurately predict the overall trend, seasonality, and dispersion of the test dataset (Figure 8). The overall fits of all models were high, and the RMSE values increased in the short test time period. The hybrid model performed equally well in describing *V. parahaemolyticus* concentrations in the training dataset and predicting *V. parahaemolyticus* concentrations in the test dataset. This model contains a minimum number of environmental variables, photoperiod and calendar day of the study, and provides a good fit for capturing and predicting the seasonality, trend and dispersion of *V. parahaemolyticus* concentration during the study period.

## 4. Discussion

The intrinsic link that *V. parahaemolyticus* has with coastal ecosystems has been well studied and characterized. Previous studies have provided many useful site- and time-specific descriptive models for describing *V. parahaemolyticus* concentration dynamics. However, few of them have been evaluated for their ability to forecast *V. parahaemolyticus* dynamics, or to be generalizable and transferable to other geographic areas or time periods. A wide range of environmental conditions and ecological interactions have been reported to influence, or at least correlate with, *V. parahaemolyticus* concentrations including water temperature, salinity, inorganic and organic nutrients, suspended solids-turbidity, chlorophyll-*a* and plankton levels, light availability, and meteorological conditions [4,5,16,17,27,29,36,38,52,53,54,55,56,57,58,59,60,61,62]. The temporal and spatial data analysis methods vary greatly in these studies, from simple correlation to more complex models [31]. These have often included the application of multiple regression analysis to characterize and model the interactions between multiple environmental parameters and *V. parahaemolyticus* levels [5,18,28,36,63,64], even though they have not been useful for forecasting *V. parahaemolyticus* dynamics and risk conditions. Based on clearly observable aspects of the *V. parahaemolyticus* concentration data for this study and some initial analyses, the combination of models applied here incorporate seasonality, trend and dispersion concepts to characterize *V. parahaemolyticus* dynamics and accurately predict *V. parahaemolyticus* concentrations. Model accuracy is in part a function of using variables that are known and consistent such as photoperiod or day of the year that are ecologically interpretable, but stable for effective *V. parahaemolyticus* forecasting. This approach of seasonality and trend analysis has the potential to be transferable for developing similar forecasting models patterns of *V. parahaemolyticus* dynamics in other locations.

*V. parahaemolyticus* concentrations in the GBE during this study followed the same pattern each year as concentrations increased rapidly each springtime as water temperatures increased, and after peak concentrations during the warmest summer conditions, decreased as water temperatures decreased in the fall each year. Such seasonality, where regular and predictable changes in environmental and climatic conditions re-occur every calendar year, tends to become more pronounced with increasing distance from the equator and is largely due to extreme temperature variation driven by variable photoperiod [65]. Water temperature accounted for approximately 48.1% of the variation observed in *V. parahaemolyticus* concentrations in this study, similar to what has been observed globally and especially in highly seasonal, temperate water regions [27,28,60]. Thus, seasonality is a significant aspect of *V. parahaemolyticus* concentration dynamics in temperate coastal areas like New Hampshire and the Northeast US. 

Photoperiod and harmonic regression models along with correlation analysis showed that *V. parahaemolyticus* concentration, water temperature, dissolved oxygen, pH, salinity and chlorophyll-*a* are significantly related to variables that mirror seasonal patterns in the GBE. Likewise, these variables accurately estimate *V. parahaemolyticus* concentrations in oysters. The synchronized seasonal periodic oscillation is one probable explanation for why regression modeling favors water temperature as the most significant model parameter. A complex combination of biological and physical environmental variables certainly drives *V. parahaemolyticus* population dynamics. However, many of these environmental variables are, in turn, driven mainly by seasonal temperature. Therefore, the variability they contribute to *V. parahaemolyticus* concentrations is not significantly different than what is provided by water temperature. For example, dissolved oxygen was negatively correlated with *V. parahaemolyticus* concentrations, similar to what has been previously reported [20] and was the second strongest variable, estimating over 32% of the variability in *V. parahaemolyticus* over the course of the study in the GBE. Since *V. parahaemolyticus* is a facultative anaerobe, this finding has the potential to elucidate important biological dimensions of the ecology of *V. parahaemolyticus*. Water temperature is a dominant driver of dissolved oxygen concentrations so collinearity between these variables is likely. In addition, because of the constraints of mathematical modeling, well-fit models are not necessarily mechanistically or ecologically descriptive [66] and, in this case, dissolved oxygen was omitted from model development to avoid multicollinearity in favor of water temperature as a stronger model variable.

Salinity and water temperature are both seasonally variable parameters that, together are the most commonly cited environmental drivers of *V. parahaemolyticus* concentration variation [16,31,67]. Salinity was a significant predictive parameter for *V. parahaemolyticus* concentration in this study, though the significance of salinity was dependent on the time interval (2007–2013 versus 2014–2016) of the data and the trend adjustment in the model (Table S2). This type of variability has also been observed in risk assessment [68,69] and in previous studies where salinity sometimes shows a strong positive correlation with *V. parahaemolyticus* [5,6,25,63], whereas for others [28,53,60,70], salinity and *V. parahaemolyticus* dynamics do not correlate. Thus, the finding that salinity and other variables reported to be significant in other *V. parahaemolyticus* concentration models were not included in this study’s final model may be, at least in part, a function of both the specific conditions at this study site and time period and a function of the in-depth statistical approach used.

Though most studies find little to no correlation between pH and *V. parahaemolyticus* concentration [20,21,28], non-linear regression and correlation analysis identified pH as an important parameter for the predictive models in the GBE. Loess smoothing highlighted the marked non-linearity of the relationship between pH and *V. parahaemolyticus* concentrations and suggested a biological optimum/optimal range for pH where *V. parahaemolyticus* concentrations decreased as pH increased or decreased relative to pH 7.8. For the purposes of optimal model development, a new pH variable was constructed by reparametrizing the measurements to create a linear response in *V. parahaemolyticus* as pH measurements moved from the optima of 7.8. An optimal pH of 7.8 is near the pH (8.5) of alkaline peptone water medium used to optimally enrich for *Vibrio* species [42] and has also been suggested as an optimal pH by laboratory-based observations [71]. Wong et al., (2015) [72] found that exposure to more acidic environments tended to reduce cell density and cause stress responses in *V. parahaemolyticus*. In this study, we observe that pH measurements in the GBE appeared to become less variable and more basic in recent years, which was also reported by Lopez-Hernandez et al., (2015) [5]. Thus, going beyond simple linear regression and including the use of non-linear analysis reveals pH as an important and ecologically linked variable to explain *V. parahaemolyticus* population dynamics. 

In other studies [36,60,63], variables other than salinity and pH were significant for estimating *V. parahaemolyticus* concentrations in univariate regression. However, in this study, they provided an insignificant amount of improvement to a multiple regression model that included water temperature. For example, chlorophyll-*a*, considered a proxy measurement for phytoplankton abundance [21,31], was significantly related to *V. parahaemolyticus* concentrations in correlation and univariate regression, but it was not significant in a multiple regression model that included water temperature. Chlorophyll-*a* was thus omitted from further model development because it did not contribute additional information in describing *V. parahaemolyticus* variation. Many studies have suggested an important ecological interaction between *V. parahaemolyticus* and plankton [16,27,57,63,69,73,74], and though chlorophyll-*a* was not included in the multiple regression models, we have also conducted a parallel study to explore the relationship between *V. parahaemolyticus* and plankton species across several years in the GBE [75,76] to determine covarying plankton species. These have included phytoplankton that have been reported to be significantly associated with *V. parahaemolyticus* elsewhere [77,78] that could provide more in-depth insight into the importance of phytoplankton and the proxy chlorophyll-*a* to the *V. parahaemolyticus* concentration dynamics observed in the GBE.

Approximately half of the variability of *V. parahaemolyticus* in the GBE could be predicted using the contribution of photoperiod (in hours), sine and cosine of the day of the study in harmonic regression, and the day of the study. Even though the model consisting solely of environmental variables was potentially more ecologically informative, the trend and seasonality variables of calendar day of the study, photoperiod, sine and cosine were more stable to estimate and predict the patterns of seasonality and trend of increasingly high concentrations over time in *V. parahaemolyticus* than salinity and to a lesser degree pH and do not require in situ measurements. Additionally, evaluation of the environmental model for its forecasting ability highlighted that some evaluation measures were discordant, while the harmonic regression and photoperiod model goodness-of-fit and forecasting error were in agreement. This highlights that though multiple evaluation measures can cause complexities in model selection, in this study the model with conflicting evaluation measures may indicate underlying issues, while the models where evaluation measurements were in agreement provided stronger prediction accuracy. Harmonic regression analyses also lead to identification of the day of year for peak *V. parahaemolyticus* concentration that occurs in mid-August (day 222 ± 5 days) that followed the peak timing of water temperature (213 ± 2), while the longest day of the year is 21 June (day 170). This highlights a loading, or hysteresis in the system and provides the basis for understanding the ‘fall shoulder’ of elevated concentrations of *V. parahaemolyticus* that extend into the late September. 

Peak timing was used to assess each environmental variable individually to detect how environmental variables may contribute to the development of ideal conditions for *V. parahaemolyticus*. Data in this study were collected either monthly or biweekly, while *V. parahaemolyticus* replicates every eight minutes under ideal conditions. In this instance, the accurate detection of lagged effects on *V. parahaemolyticus* would require more frequent sampling and fine-scale temporal resolution. Due to this level of biological complexity and the irregular temporal intervals of the data in our study, the mean from 12 h proceeding collection was used for regression with environmental variables and peak timing was used to assess temporally how each environmental variable may contribute to the development of ideal conditions for *V. parahaemolyticus*. Using this approach, we determined that significant predictive variables peak in advance of *V. parahaemolyticus* potentially contributing to a hysteresis or loading of the systems, setting up conditions that are optimal for *V. parahaemolyticus*. Davis et al., 2019 [79], recently reported that environmental variables approximately one month proceeding collection were significant to predicting *V. parahaemolyticus* concentrations in the Chesapeake Bay suggesting they might also be observing this type of lagged effect from a loading of the system. The application of harmonic regression and peak timing here demonstrates how biological complexities and limitations of sampling frequency can be overcome while also providing the resolution to detect temporal patterns between dependent and independent variables. The determination of peak timing is also a potentially important tool for forecasting the commonly observed mid-summer peaks in illnesses in the Northeast US [80]. 

A major characteristic of the *V. parahaemolyticus* concentration data is their wide dispersion. The comparison between Gaussian and negative binomial GLMs determined that the dispersion of *V. parahaemolyticus* concentrations, especially the extreme high concentrations, was best fit by the negative binomial model, as it can better account for the wide range of *V. parahaemolyticus* concentrations (0.3 to 4600 MPN/g) observed annually in the GBE. Effective risk models, with negative binomial regression as an essential model attribute, developed to predict the increasing and more dispersed *V. parahaemolyticus* concentrations will become more important as global warming and other climate and ecosystem changes will probably cause increased concentrations and persistence of *V. parahaemolyticus* in temperate coastal areas [8,81,82,83] with a likely increase in public health risks.

Model evaluation, estimations, and predictions illustrate how each model provides fit and prediction ability of the variability in *V. parahaemolyticus* concentration observed over the course of the study. Though a forecasting model consisting of environmental variables could be more appealing because of its ecological interpretability, there are potential limitations to models that rely solely on environmental predictors. For example, it is unlikely that a well-fit model can contain all the environmental variables that effect *V. parahaemolyticus* given its ecological complexity and the collinearity between seasonal-driven variables that relate to *V. parahaemolyticus* dynamics. Further, the strength of environmental variables to predict *V. parahaemolyticus* over time can change, as was observed in the interaction between pH and salinity between time intervals. Additionally, salinity became insignificant when the model was adjusted for a linear trend. The negative binomial harmonic regression and hybrid models fit the seasonality and trend features, and account well for the dispersion of *V. parahaemolyticus.* All models demonstrated good forecasting ability. Importantly, these models also enabled the determination of key characteristics of *V. parahaemolyticus* in the GBE including peak timing and a seasonal loading contributing to prolonged elevated concentrations that last into fall months. The hybrid model provides the optimal level of ecological interpretability a reasonable ability to capture the dynamics of *V. parahaemolyticus* concentrations in oysters in the GBE, and a stable platform for forecasting *V. parahaemolyticus* concentrations in coming seasons. Thus, the use of both significant environmental variables and stable parameters in the hybrid negative binomial regression model lead to successful forecasting model development that captures seasonality, temporal trends, and the high degree of data variability and dispersion.

The increased incidence of illnesses caused by *V. parahaemolyticus* infections in the Northeast US has co-occurred with increases in regional surface water temperatures and other environmental parameters, as shown in this study, suggesting an increase in the presence of pathogenic *V. parahaemolyticus* strains and/or population evolution [13,14]. The model approach developed in this study illustrates how characteristics of *V. parahaemolyticus* dynamics can be captured as environmental conditions continue to become more favorable for the pathogen to enable accurate prediction of public health risk to shellfish consumers and recreational users of coastal waters. This information, coupled with recent advances [13,14,19] that improve detection methods for endemic and invasive pathogenic *V. parahaemolyticus* sequence types (ST) in the Northeast, could be useful for shellfish harvest management in the Northeast US based on this new improved and integrated capacity to forecast concentration dynamics of both total and pathogenic *V. parahaemolyticus* populations and potential disease outbreak risks. The developed modeling approach also has the potential to inform more in-depth mechanistic studies in order to gain a better understanding of the ecology of *V. parahaemolyticus* and other water-borne pathogens. 

## 5. Conclusions

This study suggests that transferable models can be developed for forecasting public health risks related to *V. parahaemolyticus* concentrations in shellfish. Ecological monitoring data and statistical modeling are necessary to effectively characterize relationships between ecological variables and *V. parahaemolyticus* concentrations. From among many ecological variables, easy to measure water temperature and pH were all that was required when combined with seasonality and trend variables within hybrid statistical models to capture both long-term increasing trends for *V. parahaemolyticus* concentrations and to provide capacity for forecasting *V. parahaemolyticus* concentrations. The determination of peak timing is useful for assessing how each environmental variable may contribute to the development of optimal conditions for *V. parahaemolyticus*. This approach may be best applied in temperate, seasonally driven regions like the Northeast, US, as it relies on characteristics of *V. parahaemolyticus* ecology that are shared by most temperate regions. 

## Figures and Tables

**Table 1:** Trend and seasonality estimates detected by Model 1 and Model 2 for *V. parahaemolyticus* concentrations and environmental variables (Model 1, top and Model 2, bottom).

Variable ^a^	Coefficients ^b^		Standard Error		*r* ^2^	Deviance	AIC	Peak Timing ^c^
	Trend	Seasonality	Trend	Seasonality				
*Vp* (MPN/g)	0.0005 ***	0.57 ***	0.0001	0.11	0.19	0.21	673.4	
	0.0006 ***	−2.87 *** −3.66 ***	0.0001	0.34 0.33	0.50	0.51	597.4	222 ± 5
Water Temperature (°C)	<0.001	2.01 ***	<0.001	0.15	0.53	0.54	774.1	
	0.002 *	−5.81 *** −10.22 ***	<0.001	0.24 0.23	0.93	0.93	497.9	213 ± 2
Dissolved Oxygen (mg/L)	<0.001	−0.31 ***	<0.001	0.05	0.22	0.23	441.5	
	<0.001	1.45 *** 1.91 ***	<0.001	0.15 0.14	0.58	0.59	352.0	220 ± 6
Salinity (ppt)	0.001 ***	−0.19	0.0003	0.20	0.12	0.13	849.4	
	0.002 ***	−4.06 *** −1.77 **	0.0003	0.76 0.72	0.26	0.28	825.5	251 ± 18
pH	<0.001 ***	−0.02 *	<0.001	0.01	0.08	0.10	19.9	
	<0.001 ***	−0.06 0.03	0.006	0.05 0.05	0.09	0.11	20.9	298 ± 98
Turbidity (NTU)	−0.02 ***	3.93	0.007	4.10	0.06	0.09	1723.6	
	−0.02 ***	−6.34 −9.83	0.007	16.77 15.87	0.06	0.08	1716.5	135 ± 111
Chlorophyll-*a* (µg/L)	−0.0002	0.62 ***	0.005	0.0002	0.09	0.10	775.3	
	<0.001	0.11 −2.02 ***	<0.001	0.65 0.61	0.09	0.10	778.2	180 ± 37
Total Dissolved Nitrogen (mg/L)	<0.001 ***	−0.008 *	<0.001	0.005	0.15	0.16	−229.0	
	<0.001 ***	0.02 0.04 *	<0.001	0.02 0.02	0.15	0.17	−228.2	206 ± 45
Rainfall (mm)	<0.001	0.01 *	<0.001	<0.001	0.01	0.02	−76.7	
	<0.001	−0.03 −0.07 **	<0.001	0.001 <0.001	0.01	0.04	−74.6	209 ± 38

^a^ Variables are shown for Model 1, top row and Model 2, two bottom rows for sine and cosine terms; ^b^ the significance of coefficients is indicated as *** 0.001, ** 0.01, and * 0.1; ^c^ peak timing estimates are represented by the mean and standard error values; for two parameters, dissolved oxygen (DO) and total dissolved nitrogen (TDN), the estimates reflect the seasonal nadir. AIC, Akaike’s Information Criterion.

**Table 2:** Trends of the frequency of days when *V. parahaemolyticus* concentrations, water temperature and salinity exceeded the 75th percentile of data and pH data were within the 25th to 75th percentile range in GBE during the period 2007 to 2016.

Year	*V. Parahaemolyticus*		Salinity		TDN		pH	
	75th Percentile						25th and 75th Percentile	
	220 MPN/g		27 ppt		0.27 mg/L		7.56–7.88	
	*n*	%	*n*	%	*n*	%	*n*	%
2007	2/17	11.8%	196/488	40.2%	6/17	35.3%	215/488	44.1%
2008	2/18	11.1%	10/465	2.2%	0/18	0.0%	148/465	31.8%
2009	1/11	9.1%	18/463	3.9%	1/11	9.0%	173/449	38.5%
2010	3/14	21.4%	58/451	12.9%	0/14	0.0%	157/451	34.8%
2011	0/9	0.0%	46/377	12.2%	0/9	0.0%	102/430	23.7%
2012	3/7	42.9%	135/475	28.4%	0/7	0.0%	217/447	48.5%
2013	1/6	16.7%	65/438	14.8%	3/6	50.0%	231/438	52.7%
2014	7/22	31.8%	135/432	31.3%	13/22	59.1%	277/432	64.1%
2015	8/24	33.3%	205/443	46.3%	10/22	45.5%	230/408	56.3%
2016	8/21	38.1%	266/479	55.5%	4/18	22.2%	289/465	62.1%

**Table 3:** The relationship between *V. parahaemolyticus* concentrations and environmental variables and fit improvement based on linear (Model 5) and non-linear (Model 6) regression models in GBE in 2007–2017. Positive values indicate that the measure improved in Model 6 compared to Model 5 and negative values indicate a decrease in the model evaluation measurement.

Variable	Model 5	Model 6	ΔModel 6–Model 5		
	*p*-Value	*p*-Value	$\Delta r^{2}$	ΔDeviance	ΔAIC
Water Temperature (°C)	<0.001	<0.001	0.03	0.03	8.27
Dissolved Oxygen (mg/L)	<0.001	<0.001	0.04	0.05	7.28
Salinity (ppt)	<0.001	<0.001	−0.01	0.0	0.0
pH	0.009	0.002	0.14	0.08	8.48
Chlorophyll *a* (µg/L)	0.05	0.09	0.01	0.29	0.11
Rainfall (mm)	0.03	0.02	0.04	0.04	−6.31
Turbidity (NTU)	0.27	0.48	0.01	0.25	0.43
Total Dissolved Nitrogen (mg/L)	0.38	0.31	0.02	0.03	3.20

**Table 4:** The sequential building of multiple regression models for *V. parahaemolyticus* concentrations in oysters using Gaussian (GLM-G) and negative binomial (GLM-NB) models (Models 7, 8, 9).

Model Composition ^a^	Coefficients	St. Error	Deviance	AIC	Coefficients	St. Error	Deviance	AIC
Model 7 GLM-G					GLM-NB			
1. Temperature Salinity	0.34 *** 0.12 **	0.03 0.03	0.54	586.9	0.34 *** 0.13 ***	0.03 0.03	0.48	1533.4
2. Temperature C-pH ^b^	0.37 *** −4.73 ***	0.03 0.94	0.57	583.1	0.41 *** −5.52 ***	0.03 0.91	0.51	1521.6
3. Temperature C-pH Salinity	0.35 *** −3.93 *** 0.07 **	0.03 0.99 0.03	0.59	572.5	0.34 *** −4.38 *** 0.07 **	0.02 0.93 0.02	0.53	1518.3
4. Temperature C-pH Salinity C-pH*Salinity	0.35 *** 4.61 0.11 *** −0.41 ***	0.02 3.35 0.04 0.16	0.61	567.8	0.34 *** 5.52 * 0.10 ** −0.53 **	0.02 0.03 2.97 0.14	0.57	1507.1
Model 8 GLM-G					GLM-NB			
1. Trend Photoperiod Temperature C-pH	0.0003 ** −0.35 ** 0.46 *** −3.77 ***	0.0001 0.11 0.04 0.95	0.62	564.4	0.0003 *** −0.32 *** 0.43 *** −4.52 ***	0.0001 0.09 0.03 0.86	0.58	1501.9
2. Trend Photoperiod Temperature C-pH Salinity	0.0002 ** −0.32 ** 0.44 *** −3.77 *** 0.02	0.001 0.11 0.04 0.99 0.06	0.62	565.9	0.0003 *** −0.32 *** 0.43 *** −4.48 *** −0.004	0.0001 0.13 0.04 0.89 0.03	0.58	1503.9
Model 9 GLM-G					GLM-NB			
1. Trend Sin(.) Cos(.) Temperature C-pH	0.0003 ** 0.07 1.47 0.50 *** −3.78 ***	0.0001 0.69 1.12 0.11 0.96	0.62	566.3	0.0003 *** −0.28 0.79 0.41 *** −4.49 ***	0.0001 0.56 0.91 0.09 0.87	0.58	1504.2
2. Trend Sin(.) Cos(.) Temp C-pH Salinity	0.0003 ** 0.15 1.47 0.49 *** −3.60 *** 0.03	0.0001 0.69 1.12 0.11 0.99 0.04	0.62	567.7	0.0003 *** −0.31 0.77 0.41 *** −4.61 *** −0.006	0.0001 0.57 0.91 0.09 0.89 0.03	0.58	1506.2

^a^ The significance of coefficients is indicated as *** 0.001, ** 0.01, and * 0.1; ^b^ C-pH data were treated as reparametrized C-pH variables.

**Table 5:** The performance of three selected models: environmental model (Model 7.4), hybrid model (Model 8.1), and harmonic regression model (Model 9.1) for three time periods: full (P1), training (P2), and testing (P3) intervals.

Model	Variable ^a^	Time Interval		
		P1	P2	P3
Model 7.4	Coefficient: Temperature	0.34 ***	0.37 ***	0.31 ***
	Salinity	0.10 ***	0.08 **	0.24 **
	C-pH	5.51 *	5.12	266.01 ***
	Salinity*C-pH	−0.53 ***	−0.53 ***	−11.01 ***
	*r* ^2^	0.54	0.58	0.57
	Deviance	0.57	0.58	0.54
	RMSE	1.91	1.79	1.96
Model 8.1	Coefficient: Trend	0.0003 ***	0.0003	0.0007
	Photoperiod	−0.31 ***	−0.28 **	−0.48 **
	Temperature	0.43 ***	0.45 ***	0.44 ***
	C-pH	−4.51 ***	−4.32 ***	-5.10
	r2	0.61	0.57	0.61
	Deviance	0.58	0.59	0.53
	RMSE	1.85	1.81	1.92
Model 9.1	Coefficient: Trend	0.0004 ***	0.0004 *	0.0008
	Sin(.)	−0.41	−1.88 *	1.72 *
	Cos(.)	0.63	−1.54	4.66 **
	Temperature	0.40 ***	0.29 **	0.74 ***
	C-pH	−4.30 ***	−4.20 ***	1.60
	*r* ^2^	0.61	0.55	0.63
	Deviance	0.58	0.60	0.54
	RMSE	1.81	1.82	1.83

^a^ The significance of coefficients is indicated as *** 0.001, ** 0.01, and * 0.1.

## Supplementary Materials

The following are available online at https://www.mdpi.com/1660-4601/16/22/4341/s1: Table S1: Trend and seasonality estimates detected by Model 3 and Model 4. Table S2: Stepwise Development of Models 6 and 7.
