# Supplementary material for: Forecasting Seasonal Vibrio parahaemolyticus Concentrations in New England Shellfish
Source: Int J Environ Res Public Health. 2019 Nov 7;16(22):4341. doi: 10.3390/ijerph16224341 (PMC6888421; doi:10.3390/ijerph16224341)
Supplement: Supplementary file 1 [file ijerph-16-04341-s001.pdf]

Table S1. Trend and seasonality estimates detected by Model 3 and Model 4 for *V. parahaemolyticus* concentrations and environmental variables (Model 3, top and Model 4, bottom).

Variable [a]	p-value	r^2	Deviance	AIC
Vp (MPN/g)	<0.001<br>0.002	0.24	0.27	673.4
	<0.001<br><0.001<br><0.001	0.48	0.49	582.65
Water Temperature (°C)	0.11<br><0.001	0.56	0.59	772.1
	0.11<br><0.001<br><0.001	0.93	0.93	496.1
Dissolved Oxygen (mg/L)	0.82<br><0.001	0.21	0.23	441.5
	0.87<br><0.001<br><0.001	0.58	0.59	352.0
Salinity (ppt)	<0.001<br>0.45	0.38	0.42	805.6
	<0.001<br><0.001<br>0.01	0.48	0.51	781.2
pH	<0.001<br>0.16	0.18	0.23	10.1
	<0.001<br>0.54<br>0.41	0.18	0.24	12.9
Turbidity (NTU)	0.04<br>0.11	0.03	0.50	1713.6
	0.03<br>0.37<br>0.38	0.03	0.05	1715.9
Chlorophyll-*a* (µg/L)	<0.001<br><0.001	0.24	0.30	758.3
	<0.001<br>0.67<br><0.001	0.24	0.30	759.0
Total Dissolved Nitrogen (mg/L)	<0.001<br>0.01	0.46	0.49	-289.3
	<0.001<br>0.14<br>0.002	0.47	0.50	-289.9
Rainfall (mm)	0.37<br>0.07	0.03	0.06	-76.4
	0.29<br>0.24<br>0.02	0.04	0.08	-76.9

Table S2. Model variables, their significance and model fit evaluation for models based on combinations of photoperiod, day of study, harmonic regression and environmental variables. A. Gaussian. B. Negative binomial regression.

A. Model-Gaussian	Coefficient	r^2	Deviance	AIC
Model 1	0.57*** 0.0002***	0.19	0.20	666.4
Model 1 + sine + cosine	-4.1 0.0006*** -0.53 -1.6	0.51	0.52	596.9
Model 1 + Temp	-0.37** 0.0004*** 0.47***	0.57	0.57	577.6
Model 1 + Temp + Sal	-0.30** 0.0004** 0.44*** 0.06	0.57	0.58	577.0
Model 1 + Temp + C-pH	-0.3** 0.0003** 0.46*** -3.8***	0.61	0.61	564.4
Model 1 + Temp + C-pH + Sal	-0.3** 0.0002** 0.44*** -3.6** 0.02	0.61	0.62	565.9
Model 2+ Temp	0.0004*** 0.06 1.51 0.51***	0.56	0.57	579.7
Model 2 + Temp + Sal	0.0003** 0.23 1.5 0.5*** 0.06	0.57	0.58	578.9
Model 2 +Temp + C-pH	0.0003** 0.07 1.4 0.50*** -3.8***	0.61	0.61	566.3
Model 2+ Temp + C-pH + Sal	0.0003** 0.15 1.5 0.49*** -3.6** 0.03	0.60	0.62	567.7
B. Model-Negative binomial				
Model 1	0.6*** 0.0006***	0.19	0.17	1619.5
Model 1 + sine + cosine	-6.2** 0.0005*** 0.6 -0.3***	0.49	0.52	1532.8
Model 1 + Temp	-0.32** 0.0004*** 0.4***	0.56	0.53	1521.3
Model 1 + Temp + Sal	-0.27** 0.0004** 0.4*** 0.05*	0.56	0.53	1521.1
Model 1 + Temp + C-pH	-0.32*** 0.0003***; 0.4 -4.5	0.61	0.58	1501.9
Model 1 +Temp + C-pH + Sal	-0.32*** 0.0003*** 0.4*** -4.5*** -0.004	0.61	0.58	1503.9
Model 2 + Temp	0.0003*** -0.3 0.8 0.4***	0.55	0.52	1523.8
Model 2 + Temp + Sal	0.0003*** 0.03 1.1 0.4*** 0.05*	0.56	0.53	1523.5
Model 2 +Temp + C-pH	0.0003*** -0.3 0.8 0.4*** -4.5***	0.61	0.58	1504.2
Model 2 + Temp + C-pH + Sal	0.0003*** -0.3 0.8 0.4*** -4.6*** -0.006	0.61	0.58	1506.2

significance of coefficients indicated as *** 0.001, ** 0.01, * 0.1.
